# Targeting Excitatory Glutamate Receptors for Morphine Tolerance: A Narrative Review

**DOI:** 10.1111/cns.70468

**Published:** 2025-06-08

**Authors:** Min Huang, Limin Luo, Wenying Wang, Hao Xu, Moxi Chen, Xiaqing Ma, Tao Xu

**Affiliations:** ^1^ Department of Anesthesiology Sixth People's Hospital Affiliated to Shanghai Jiao Tong University School of Medicine Shanghai China; ^2^ Department of Anesthesiology First Affiliated Hospital of Nanjing Medical University Nanjing China; ^3^ Department of Anesthesiology Affiliated Hospital of Nantong University, Medical School of Nantong University Nantong China; ^4^ School of Rehabilitation Science Shanghai University of Traditional Chinese Medicine Shanghai China

**Keywords:** drug tolerance, excitatory amino acids, glutamate receptors, opioid analgesic

## Abstract

**Background:**

Opioids remain a primary treatment for moderate‐to‐severe chronic pain, but prolonged use frequently induces analgesic tolerance. Excitatory glutamate receptors, ubiquitous in the central and peripheral nervous systems, are pivotal in physiological and pathological processes and are now recognized as significant contributors to opioid tolerance development.

**Methods:**

We comprehensively analyzed experimental data from our team's intensive investigations over the past 10 years, alongside relevant peer‐reviewed studies on glutamate receptor mechanisms in opioid tolerance.

**Results:**

Excitatory glutamate receptors (including NMDA, AMPA, and metabotropic subtypes) fundamentally regulate neuroadaptations underlying morphine tolerance. Our work and others' demonstrate their involvement in synaptic plasticity, neuroinflammation, and downstream signaling pathways that drive tolerance.

**Conclusions:**

Targeting excitatory glutamate receptors presents a promising therapeutic strategy for mitigating opioid tolerance. Future research should prioritize elucidating receptor‐specific mechanisms, peripheral‐central nervous system crosstalk, and translating preclinical findings into clinically viable.

## Graphical abstract

Schematic diagram for the excitatory glutamate receptors for morphine tolerance and drugs targeting glutamate receptors in morphine tolerance. This figure illustrates the main mechanisms by which the major ionic and metabotropic glutamatergic receptors are involved in the process of morphine tolerance. In addition, several drugs that may alleviate morphine tolerance by acting on glutamatergic receptors are presented.

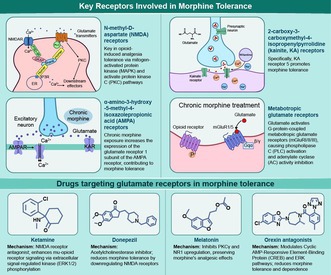

## 1 Introduction

Although new analgesics have emerged recently, opioids remain a preferred option for managing both acute and chronic pain. However, their reduced efficacy due to long‐term use poses a major problem hindering their use in clinical scenarios. Akil first published their findings in Science in 1991 that MK‐801, a noncompetitive NMDA receptor antagonist, can preserve the acute analgesic effects of morphine while significantly alleviating the chronic effects of morphine [1]. In addition to MK‐801, in their subsequent studies, they demonstrated that several noncompetitive NMDA receptor antagonists, including ketamine, dextrorphan, and phencyclidine, exhibited significant retarding properties of opioid tolerance [2]. Similar results have been obtained by several other laboratories using NMDA receptor competitive antagonists such as NPC17742 [3] and LY274614 [4], and even NMDA receptor glycine modulatory site antagonists such as ACEA‐1328 [5]. Since then, much attention has been paid to the role of excitatory glutamate receptors in morphine tolerance. The central glutamatergic and spinal dynorphin systems are involved in opioid‐induced hyperalgesia and analgesic tolerance [6, 7]. In this narrative review, we discuss the role of glutamate receptors, including ionotropic (ligand‐gated cation channels) and metabotropic (G protein‐coupled receptor) families, in the development of morphine tolerance and highlight drugs with therapeutic potential.

**FIGURE 1:**
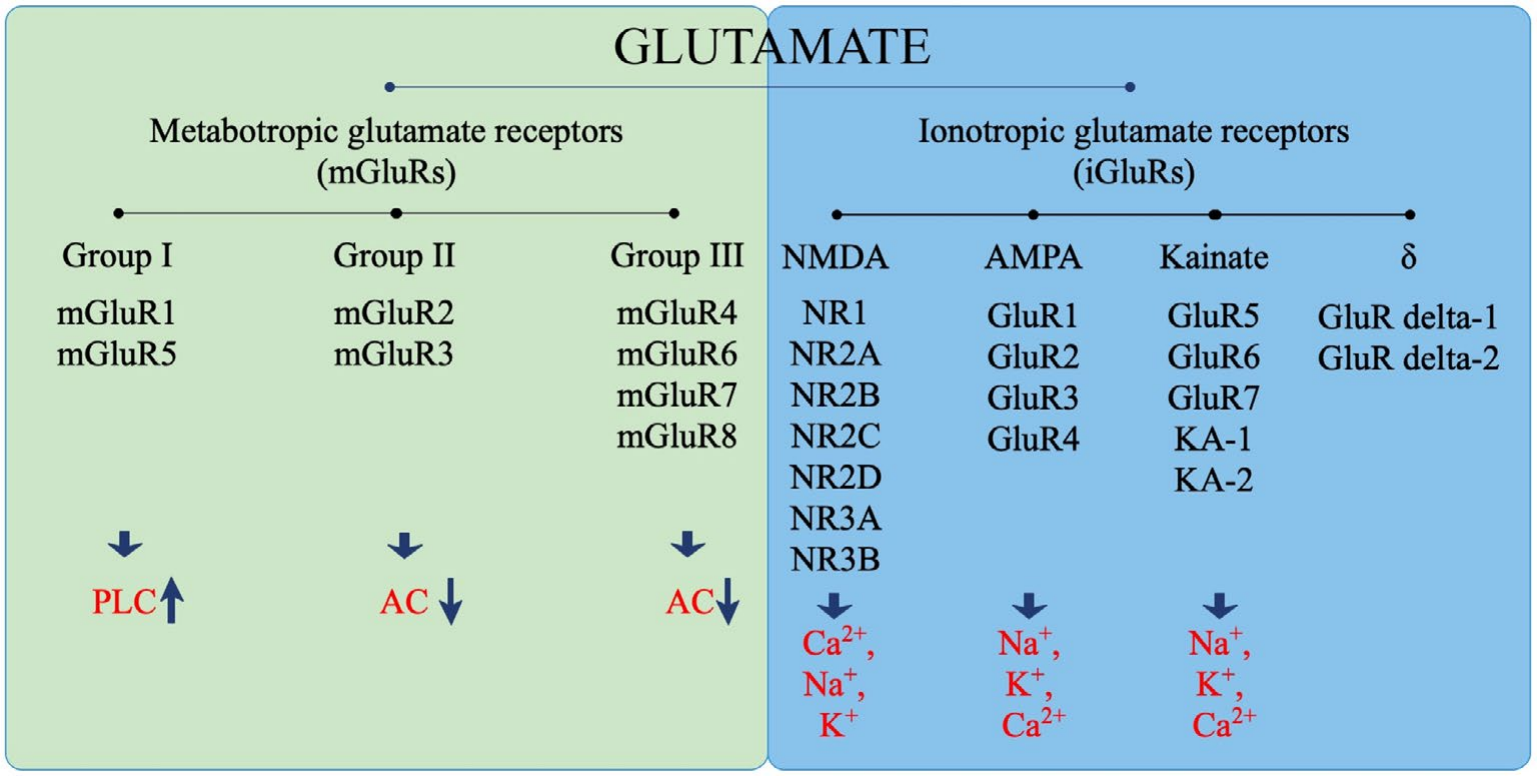
Glutamatergic receptor families can be divided into two types, metabotropic and ionotropic glutamate receptors. Glutamate activates G‐protein‐coupled metabotropic glutamate receptors (mGluRI/II/III), thereby activating phospholipase C (PLC) and inhibiting adenylate cyclase (AC) activity. Ionotropic glutamate receptors are nonselective cation channels that allow the passage of Na^+^, K^+^, and Ca^2+^_,_ thus mediating fast signaling. Upward arrows (↑) indicate activation, and vice versa, downward arrows (↓) represent inhibition.

Glutamate, the major excitatory neurotransmitter in the mammalian central nervous system (CNS), mediates its effects through ionotropic glutamate (iGluRs) and metabotropic glutamate (mGluRs) receptor families (Figure 1). The important role of glutamate receptors in the CNS has spurred extensive research into their functions in various physiological processes, including neuronal development, neurotoxicity, and neuronal plasticity. These processes explore glutamate as a key cellular substrate in learning, memory, addiction, and pain.

According to the agonists originally identified to activate each specific class of receptors, there are four classes of iGluRs: N‐methyl‐D‐aspartate (NMDA), α‐amino‐3‐hydroxy‐5‐methyl‐4‐isoxazolepropionic acid (AMPA), 2‐carboxy‐3‐carboxymethyl‐4‐isopropenylpyrrolidine (kainate, KA), and δ receptors [8]. Each iGluR is an integral membrane protein composed of four large subunits that together form a central ion channel pore. The structural components common to each subunit include an extracellular amino‐ and carboxy‐terminal domain, and a large intracellular loop between transmembrane domains 3 and 4. iGluRs are nonselective cation channels that allow the passage of Na^+^, K^+^, and Ca^2+^, facilitating rapid signaling. iGluRs play key roles in various neuroplasticity processes, such as peripheral and central sensitization, learning, memory, long‐term potentiation, and some other adaptive responses [9, 10].

Metabotropic glutamate receptors, part of the G protein‐coupled receptor superfamily, are grouped into three classes (I–III) based on the similarities in conjugation mechanism, molecular structure, sequence homology, and receptor pharmacology. The eight mGluR subtypes (1–8) identified so far are classified into groups I–III (Figure 1). Metabotropic glutamate receptors 1 and 5 are key members of the group I mGluRs, which are positively correlated with phospholipase C (PLC). This association can increase the levels of inositol‐1,4,5‐triphosphate (IP3) and intracellular Ca^2+^, and activate protein kinase C (PKC). Group II (mGluR 2 and 3) and Group III (mGluR 4, 6, 7, and 8) receptors are negatively coupled to adenylate cyclase (AC) via Gi/Go proteins, mainly expressed presynaptically, and typically inhibit the release of neurotransmitters such as glutamate and gamma‐aminobutyric acid [11, 12]. Unlike iGluRs, mGluRs regulate rather than directly mediate excitatory synaptic transmission.

## 2 Ionotropic Glutamic Receptors and Morphine Tolerance

### 2.1 NMDA Receptors

The NMDA receptor, an ion‐channel receptor, is predominantly located at excitatory synapses and is mainly distributed in the presynaptic and postsynaptic membranes of the spinal dorsal horn [13, 14, 15]. This receptor is a glutamate‐activated calcium ionophore, comprising pore‐forming structures and auxiliary subunits (glutamate 1, 2A–2D, 3A, and 3B receptors) that dictate the functional properties of native NMDA receptors [16].

Activation of NMDA receptors is primarily voltage‐dependent, and the Ca^2+^ ion currents transmitted through these receptors are pertinent for synaptic plasticity [17]. Activation removes the voltage‐dependent Mg^2+^ block, allowing rapid Ca^2+^ influx into postsynaptic secondary neurons [18]. This increased intracellular Ca^2+^ activates Ca^2+^‐sensitive intracellular signaling cascades, leading to NMDA receptor phosphorylation and PKC activation [19]. Furthermore, NMDA receptors play a key role in both the transmission and modulation of pain signaling [20, 21].

NMDA receptors are complex polymeric proteins assembled from various combinations of NR1 and NR2 subunits. The same gene encodes at least eight different NR1 subunits, whereas multiple genes within the same gene family encode four different NR2 subunits (NR2A, NR2B, NR2C, and NR2D). The physicochemical properties of most NMDARs are defined by the NR2 subunit composition [22]. Notably, the NR1 and NR2B subunits have been widely implicated in the development of morphine tolerance [23, 24]. Opioids may induce tonic activation of glutamatergic NMDA receptors at the terminals of primary afferents [25].

NMDA receptors undergo internalization through clathrin‐mediated endocytosis when activated by agonists [26, 27]. Sustained glutamate release can also activate the calcium‐dependent protease calpain, leading to postsynaptic NMDA receptor cleavage [28, 29]. Additionally, increased glutamate release with chronic morphine use may increase calcineurin activity, thereby downregulating postsynaptic NMDA receptor levels [30].

NMDA receptors in neurons co‐localize with postsynaptic density protein (PSD)‐95, a putative NMDA receptor‐anchored protein that is a core component of PSD on the excitatory synaptic membranes [31]. The number of functional NMDA receptors on the neuronal cell surface and NMDA channel opening rates were raised through PKC activity [32, 33]. Similarly, PSD‐95 promotes new NMDA receptor insertion, stabilizes receptors at the plasma membrane, and increases the rate of channel opening [34]. Importantly, PKC enhances NMDA responses and surface expression to comparable levels regardless of the presence or absence of PSD‐95 [35]. Moreover, while PKC promotes the interaction between NR2A and NR1 subunits, it does not affect the interaction between NMDA receptors and PSD‐95 in neurons. A potential explanation for this specificity is that PKC facilitates the assembly and insertion of PSD‐95, independent of NMDA receptors [36].

### 2.2 NMDA Receptors and Morphine Tolerance

The mitogen‐activated protein kinase (MAPK) signaling pathway mediates the activation of presynaptic NMDA receptors and leads to opioid‐induced analgesic tolerance [37, 38]. β‐arrestin may be involved in the rapid attenuation of opioid analgesia and development of tolerance [39, 40]. Arrestin may act as a scaffold for MAPK, enhancing NMDA activation [41]. Phosphorylated β‐arrestin bound to mu‐opioid receptor complexes negates the effects of mu‐opioid receptors and recruits MAPK signaling molecules [42]. Additionally, NMDA receptors have been implicated in upstream spinal c‐Jun N‐terminal kinase (JNK) activation in morphine antinociceptive tolerance [43]. Inhibiting the activity of extracellular regulated protein kinases 1/2 (ERK1/2), p38, or JNK at the spinal cord level can preserve the analgesic effect of morphine more effectively than abolishing morphine‐induced hyperalgesia [37].

Following NMDA receptor activation, substantial Ca2+ influx occurs through channels regulated by these receptors. This influx of intracellular Ca^2+^ is implicated in several major neuronal excitotoxicity mechanisms and synaptic disturbances, potentially contributing to PKC activation [44]. These changes promote the NMDA‐induced extension of transient membrane Ca^2+^ currents, enhancing glutamatergic transmission and inducing a postsynaptic long‐term potentiation (LTP) state. Furthermore, these effects lead to the phosphorylation of membranal mu‐opioid receptors and tolerance development [44].

Intrathecal PKC inhibitors can reverse chronic morphine exposure‐induced opioid tolerance [45], and increased PKC activity enhances the expression and modulates the channel gating of NMDA receptors [32, 46]. Chelerythrine, a membrane permeability inhibitor at the catalytic site of PKC [47], inhibits PKC activity in brain slices [48]. Incubation of morphine‐treated spinal cord slices with chelerythrine reduced the baseline frequency of spontaneous excitatory postsynaptic currents (sEPSCs) and the amplitude of monosynaptic excitatory postsynaptic currents (mEPSCs). Thus, PKC plays a key role in the long‐term opioid‐induced increases in presynaptic NMDA receptor activity in the spinal cord [38]. Moreover, PKC prompts the rapid transfer of functional NMDA receptors to the cell surface and increases NMDA receptor expression, as shown via immunofluorescence analysis of surface NR1. Thus, PKC influences synaptic plasticity by regulating the gating and trafficking of NMDA receptor channels in recombinant systems and neurons [32]. NMDA receptors activate nitric oxide synthase (NOS) excitatory signaling in response to chronic morphine treatment [49]. NMDA receptors alter NOS activity in the brain [50, 51]. Most importantly, blockers of the NMDA/NOS receptor ion channels mimic CNS disease and even interfere with the development of opioid tolerance and physical dependence [52]. Therefore, the NMDA receptor and nitric oxide (NO) pathways may interact with the development of opioid tolerance in certain disease models. Mu‐opioid receptors transiently activate the Akt‐neuronal NOS (nNOS) pathway, which contributes to sustaining the potentiation of PKC‐mediated NMDA receptor–calmodulin‐dependent protein kinase II (CaMKII) signaling [53].

Purinergic 2X (P2X) receptors, a family of ligand‐gated ion channels activated by extracellular ATP, have been implicated in the pathogenesis of various pain types, such as inflammatory pain and neuropathic pain [54, 55]. In the spinal cords of morphine‐tolerant rats, modulation of P2X receptor expression can regulate NMDA receptor expression and synaptic excitatory amino acid levels [56]. Intrathecal injection of the P2X receptor antagonist, 2’,3’ ‐O‐(2,4,6‐ trinitrophenyl) (TNP)‐ATP partially restored the analgesic effect of morphine in tolerant rats, likely via inducing the internalization of NR1 and NR2B from the synaptosome membrane into the cytoplasm of neurons, thereby reducing NMDA receptor‐mediated intracellular signaling and excitatory amino acid release [57, 58].

Long‐term morphine administration increases the levels of tumor necrosis factor (TNF)‐α, interleukin (IL)‐1β, and IL‐6, and nitration of glutamate transporter‐1 and glutamine synthetase, and expression of NR1 and NR2B [59, 60, 61]. Injection of hydrogen‐enriched saline not only attenuated morphine tolerance but also decreased proinflammatory cytokine expression and glutamate transporter‐1 and glutamine synthetase nitration. Moreover, it inhibited astrocyte activation and membrane trafficking of NMDA receptors [62]. Intrathecal administration of hydrogen preconditioning may prevent morphine tolerance by reducing neuroinflammation and NMDA receptor trafficking [62, 63].

Prolonged morphine administration reduces *NR1* expression in the lumbosacral spinal cord while conversely increasing it in the midbrain [64]. Downregulation of NR1 mRNA levels in the spinal dorsal horn was also found to correlate with morphine tolerance [65, 66]. Injecting the mGluR5 agonist 3,5‐dihydroxyphenylglycine (DHPG) into the dorsal striatum increases the phosphorylation of NR1 [67]. Moreover, the increased expression of the NMDA NR1 receptor caused by chronic morphine treatment could be inhibited by antagonizing the activation of mGluR5 [68].

Sustained stimulation of opioid receptors by endogenous opioids leads to analgesic tolerance, possibly due to changes in synaptic plasticity. Elimination of analgesic tolerance to repeated morphine administration was observed in NR2A^−/−^ mice [69]. Morphine analgesic tolerance was restored by re‐expression of *NR2A* in the periaqueductal gray matter (PAG) or ventral tegmental area of NR2A^−/−^ mice. Morphine analgesia, which was originally lacking in the mouse stress model, was also fully restored in NR2A^−/−^ mice [70]. The microinjection of NR2A small interfering RNA into the PAG significantly reversed morphine analgesic tolerance [69, 71], highlighting the potential role of the NR2A‐NMDA receptor system as a key mechanism in the development of morphine tolerance in stress model mice, though the specific molecular mechanism remains unclear. Moreover, while NR2A protein expression in the PAG is upregulated after long‐term morphine treatment [69], the expression of other NMDA receptor subunits and tolerance‐related proteins such as the μ‐opioid receptor and PSD‐95 was not detected [70, 72]. Another study demonstrated that methadone exposure in mice pretreated with morphine was able to reverse morphine tolerance by blocking several cellular hallmarks in PAG such as cAMP overshoot or NMDA receptor regulation [73]. Moreover, this effect involves the ability of methadone to promote MOPr endocytosis. It is reported that MOR and NR1 subunits associate in the postsynaptic structures of PAG neurons. Morphine can disrupt this complex by PKC‐mediated phosphorylation of the NR1 C1 segment and potentiates the NMDAR–CaMKII pathway that is implicated in morphine tolerance. Inhibition of PKC restored the MOR–NR1 association and rescued the analgesic effect of morphine. The administration of N‐methyl‐D‐aspartic acid separated the MOR–NR1 complex, increased MOR Ser phosphorylation, reduced the association of the MOR with G‐proteins, and diminished the antinociceptive capacity of morphine. Inhibition of PKA blocked these effects and preserved morphine antinociception. Thus, the opposing activities of the MOR and NMDAR in pain control affect their relation within neurons of structures such as the PAG. This finding could be exploited in developing bifunctional drugs that would act exclusively on those NMDARs associated with MORs [74].

A wealth of evidence suggests that selective antagonists of the NMDA receptor subunit NR2B have greater inhibitory activity and fewer morphine tolerance or addictive side effects than those of nonspecific antagonists [75, 76, 77, 78, 79]. For instance, ifendil, a typical NMDA receptor NR2B selective antagonist, is associated with fewer adverse effects, such as psychosis, ataxia, and sedation, than those associated with the nonselective inhibitor MK‐801 at similar doses [77]. Additionally, 4‐((1R,2S)‐3‐(4‐benzylpiperidin‐1‐yl)‐1‐hydroxy‐2‐methylpropyl)phenol (Ro‐256,981), a selective NMDA NR2B antagonist, effectively suppresses morphine tolerance in mice or rats with a low incidence of side effects [78, 79].

NMDA antagonists, competitive and noncompetitive, sustain the analgesic effects of morphine in models of morphine tolerance and neuropathic pain [80, 81]. NR2B subunit overexpression enhances inflammatory pain [82], whereas inhibition of NMDA receptor expression using antisense oligonucleotides has prevented formalin‐induced pain [83]. MK‐801 pretreatment can partially reverse the increased expression of proinflammatory cytokines in activated microglia and astrocytes in morphine‐tolerant rats, thereby relieving their branching state [84].

Other NMDA receptor agonists, such as dextromethorphan and ketamine, also have anti‐inflammatory effects [85, 86]. Dextromethorphan protects dopaminergic neurons by inhibiting the activation of microglia and reducing the release of NO, TNF‐α, prostaglandin E2, and superoxide, thereby preventing lipopolysaccharide (LPS)‐induced neurodegeneration [87, 88]. These findings provide multiple lines of evidence that NMDA receptors in the spinal dorsal horn are essential for neuroplasticity in various pain‐related behaviors, including tolerance, hyperalgesia, and allodynia [84].

## 3 Non‐NMDA Ionotropic Glutamate Receptors

### 3.1 AMPA Receptors

AMPA receptors are glutamate‐gated ion channels, including four functional subunits as glutamate receptors 1–4, which compose the core functional ion channel by combining into tetramers, and different combinations of glutamate receptors 1–4 subunits produce unique biophysical characteristics. AMPA receptors are widely distributed in the brain. As reported, AMPA receptors are essential in the regulation of fast excitatory synaptic transmission in the mammalian CNS [89, 90]. The alteration in the number, composition, and biophysical properties of postsynaptic AMPA receptors mediates synaptic strength, contributing to the formation of postsynaptic LTP, long‐term depression (LTD), and homeostatic synaptic plasticity at excitatory synapses [91, 92, 93, 94]. Such neuronal processes, like activation of neuroexcitatory mechanisms, utilize LTP, which contributes to morphine tolerance [95, 96]. Therefore, the dynamic changes of AMPA receptors are closely related to morphine tolerance by influencing postsynaptic LTP and LTD formation.

### 3.2 AMPA Receptors and Morphine Tolerance

**FIGURE 2:**
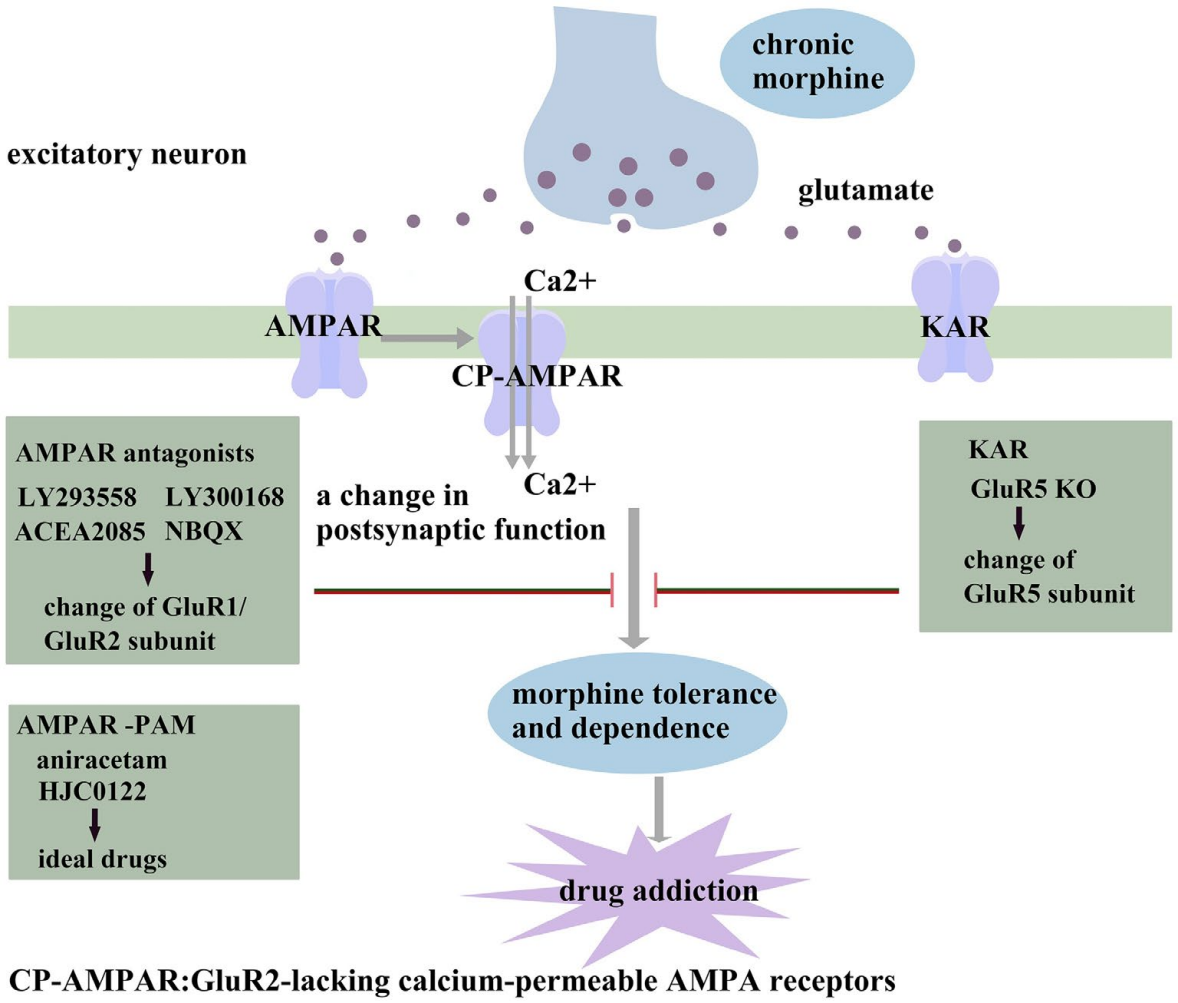
Glutamate receptor 1 and 2 subunits are important functional subunits of AMPA receptors. Glutamate receptor 2 is a critical subunit in determining mammalian AMPAR function and one of the most tightly regulated glutamate receptor subunits. Chronic morphine exposure increases the expression of the glutamate receptor 1 subunit in the CNS postsynaptic membrane, contributing to morphine tolerance. It also causes a deficiency of glutamate receptor 2 subunits of AMPARs on the CNS postsynaptic membrane, leading to the formation of the GluA2‐lacking calcium‐permeable AMPA receptor (CP‐AMPA receptor). This effect results in a massive influx of calcium ions and a change in postsynaptic function, which contribute to behavioral sensitization and morphine tolerance and dependence. Specific antagonists of the AMPA receptor, such as NBQX, can silence this pathway and reverse established morphine tolerance and dependence.

Many studies have shown that the AMPA receptor family is closely related to the development of morphine tolerance and dependence (Figure 2). Morphine tolerance and dependence are less severe in mice lacking the AMPA glutamate receptor 1 subunit, with these mice also exhibiting reduced scores for naloxone‐induced opioid withdrawal symptoms [97].

Considerable evidence shows that the AMPA receptor antagonists, such as 6‐[2‐(2H‐tetrazol‐5‐yl)ethyl]‐1,2,3,4,4a,5,6,7,8,8a‐decahydroisoquinoline‐3‐carboxylic acid (LY293558) and 5‐(4‐aminophenyl)‐N,8‐dimethyl‐8,9‐dihydro‐[1,3]dioxolo[4,5‐h][2,3]benzodiazepine‐7‐carboxamide (LY300168) can reduce morphine withdrawal symptoms and inhibit the activation of locus coeruleus neurons [98]. LY293558 and (3S,4aR,6S,8aR)‐6‐(phosphonomethyl)‐1,2,3,4,4a,5,6,7,8,8a‐decahydroisoquinoline‐3‐carboxylic acid (LY235959) have also been found to reduce the incidence of acute morphine dependence without affecting its analgesic effects (Figure 2). However, these compounds did not influence the analgesic tolerance or dependence induced by morphine pellet implantation [99].

Moreover, continuous administration of LY293558 blocked the onset of acute morphine somatic dependence in mice, delayed the development of morphine tolerance, and reversed established tolerance [100, 101]. In addition, ACEA2085, another AMPA receptor antagonist, enhanced the antinociceptive response of morphine to acute heat stimulation, even though ACEA2085 itself exhibited some antinociceptive effects [102] (Figure 2).

In mice with established tolerance, the administration of 2,3‐dihydroxy‐6‐nitro‐7‐sulfamoyl‐benzoquinoxaline‐2,3‐dione (NBQX), an AMPA receptor antagonist, did not alter behavioral sensitization. In tolerant mice, 7 consecutive days of washout reduced glutamate receptor 2 mRNA expression by 50% in the amygdala and 35% in the hippocampus, while glutamate receptor 3 mRNA expression remained unchanged in all the brain regions studied [103] (Figure 2). The reduced glutamate receptor 2 mRNA expression in the amygdala and hippocampus may lead to the formation of calcium‐permeable AMPA receptors, contributing to behavioral sensitization (Figure 2). Contrasting with NMDA receptor antagonists, which were ineffective in reducing morphine tolerance or withdrawal in 7‐day‐old model rats, AMPA receptor antagonists proved effective at all tested ages [104]. Therefore, in the development of treatments for human infants, focusing on non‐NMDA ionotropic receptors to reduce opioid tolerance and withdrawal might be more appropriate.

Aniracetam, a positive allosteric modulator of AMPA receptors (AMPA receptor‐pam), can prevent and reverse the development of acute and chronic morphine analgesic tolerance, as well as intermittent morphine dependence (Figure 2). Furthermore, HJC0122, a novel AMPA receptor‐PAM, has a higher potency in reversing morphine tolerance than that of aniracetam [105]. The reversal of opioid resistance by AMPA receptor‐PAMs may be attributed to their ability to reduce desensitization and inactivation of the receptor itself while increasing the amplitude and dynamics of AMPA receptor‐mediated synaptic currents [106, 107] (Figure 2).

AMPA receptor‐PAMs can stimulate ventilatory rhythmogenesis during respiratory suppression episodes, making these compounds ideal for the selective suppression of opiate‐induced respiratory depression [108, 109, 110]. Thus, AMPA receptor‐PAMs can be considered optimal for use with opioids due to their role in mitigating the onset of opioid tolerance and dependence and their minimal risk of exacerbating respiratory suppression. Acute treatment with TNF inhibitor etanercept in morphine‐tolerant rats prevented the upregulation of AMPA receptors, NMDA receptor subunits NR1/NR2A, and glutamate receptor 1/2, which contributed to significant analgesic effects in these animals [111].

### 3.3 KA Receptors

Current studies on the relationship between KA receptors and morphine tolerance mainly focus on the glutamate receptor 5 subtype. Some studies have shown that glutamate receptor 5 isoforms can promote the development of morphine tolerance without affecting the nociceptive threshold or the accumulation of morphine and its metabolites in the CNS [112].

#### 3.3.1 KA Receptors and Morphine Tolerance

LY293558, a glutamate receptor 2/5 preferred antagonist, blocks acute and chronic morphine tolerance [100]. However, LY293558 was originally identified as an AMPA receptor antagonist because of its higher binding affinity for glutamate receptor 2 than for glutamate receptor 5 subunits [113]. Due to the lack of an ideal drug, glutamate receptor 5 or glutamate receptor 6 knockout (KO) mice were used for related studies [114]. The results showed that glutamate receptor 5 KO mice were less likely to develop morphine tolerance after repeated subcutaneous and intraperitoneal injections of cocaine or subcutaneous implantation of morphine granules than their wild‐type littermates [112, 115, 116]. These findings shed light on the role of KA receptor subtypes containing glutamate receptor 5 in promoting morphine tolerance [112, 115, 116].

## 4 Metabotropic Glutamate Receptors and Morphine Tolerance

### 4.1 Group I Metabotropic Glutamate Receptors

In 1994, Fundytus showed that chronic intracerebroventricular co‐infusion of the mGluR antagonist (S)‐4‐carboxyphenylglycine ((S)‐4C‐PG) and systemic morphine administration attenuated the development of morphine tolerance [117]. (S)‐4C‐PG not only selectively antagonizes group I mGluR (1 and 5) but also activates group II mGluR (2 and 3). Therefore, it remains unclear whether the mechanism by which (S)‐4C‐PG delays morphine tolerance is due to its antagonism of type 1/5 mGluRs or its activation of type 2/3 mGluRs.

**FIGURE 3:**
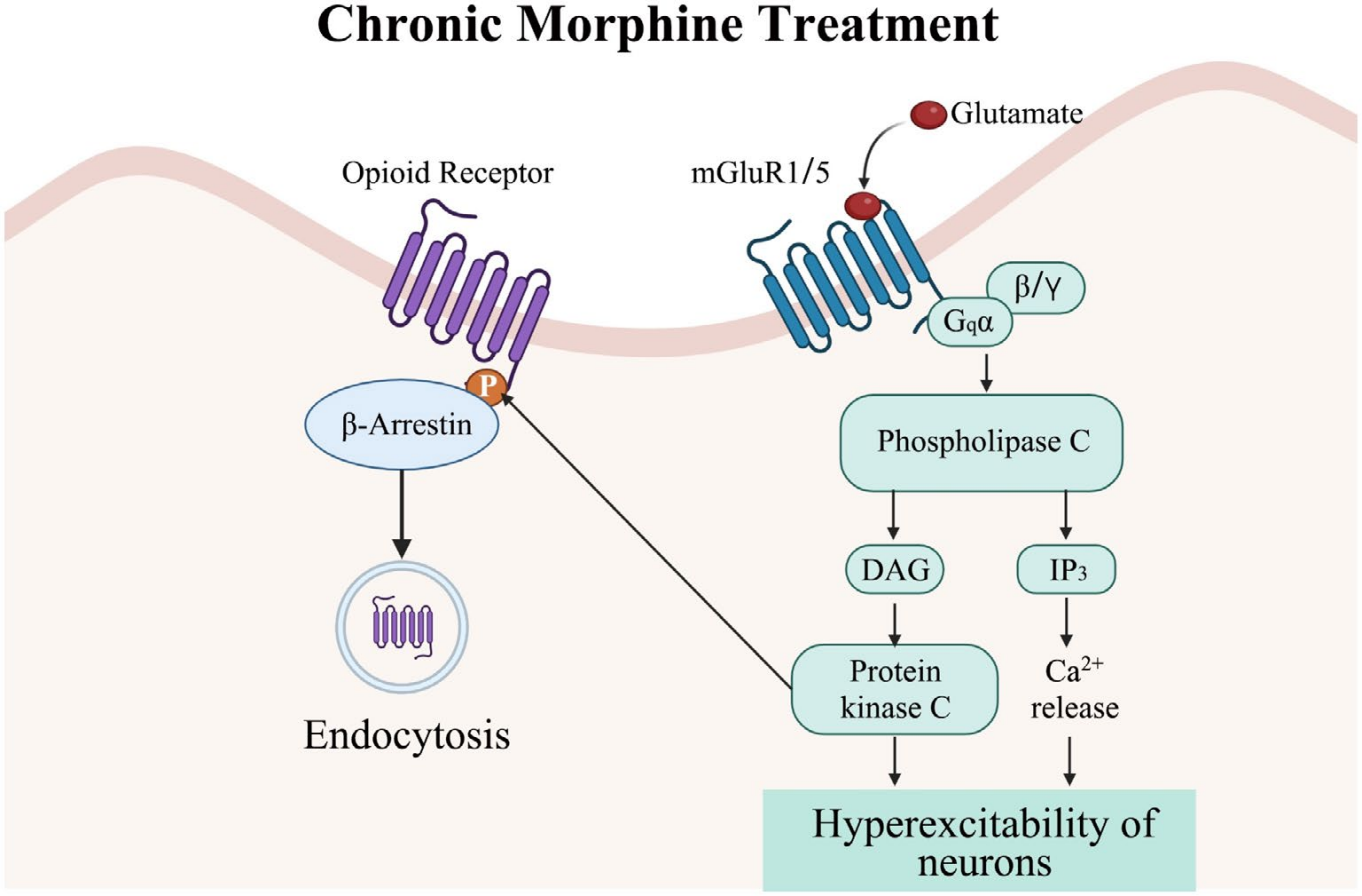
Schematic representation of possible mechanisms underlying metabotropic glutamate receptor 1/5 pathway mediation of morphine tolerance. After chronic treatment with morphine, neurons are activated, leading to overexpression of metabotropic glutamate receptor 1/5, which activates metabotropic glutamate receptor 1/5 pathways. Metabotropic glutamate receptor 1/5 is positively linked to phospholipase C (PLC), which increases inositol‐1,4,5‐triphosphate (IP3), intracellular Ca^2+^, and PKC activities. All these activities contribute to neuronal hyperexcitability, which may explain the development of morphine tolerance. Additionally, compelling evidence indicates that PKC, possibly through the direct phosphorylation of mu‐opioid receptors, contributes to morphine‐induced desensitization [123].

Extensive evidence over the years has underscored that group I mGluRs play an important role in the development of morphine antinociceptive tolerance. Metabotropic glutamate receptor 1, predominantly expressed in laminae I and II of the dorsal spinal cord, has been implicated in opioid dependence [118]. The mGluR1 receptor selective antagonist 7‐(hydroxyimino) cyclopropa[b]chromen‐1a‐carboxylate ethyl ester (CPCCOEt) and the mGluR5 selective antagonist 2‐methyl‐6‐(phenylethynyl) pyridine hydrochloride (MPEP) can partially but significantly reverse morphine tolerance. However, their combined administration fully reversed this tolerance [119]. Co‐administration of the group I mGluR antagonist (RS)‐1‐aminoindan‐1,5‐dicarboxylic acid (AIDA) or MPEP with morphine reduced chronic morphine‐induced upregulation of NR1 expression in the spinal dorsal horn and progression of tolerance [68]. The combined infusion of MPEP and morphine preserved the analgesic effect of morphine, delayed the development of tolerance, and partially inhibited the upregulation of nNOS protein in the spinal dorsal horn [120]. Conversely, the specific mGluR5 agonist CHPG exhibited opposite effects. Activation of mGluR5 mobilizes intracellular Ca^2+^ release and activates PKC, which inhibits morphine antineurotoxic stimuli [121]. The reduced morphine tolerance in *mGluR1* knockdown mice is related to a reduced potential to stimulate the increase of PLC, thereby reducing Ca^2+^, IP3, PKC, and other related intracellular signals [122] (Figure 3). Our previous study revealed that chronic morphine‐induced analgesic tolerance and hyperalgesia were significantly reduced in mGluR5 knockout (KO) mice, and chronic morphine‐induced NR2B phosphorylation levels were decreased, although the total NR2A expression level remained unchanged [124].

Cellular experiments indicate a physical connection between the μ‐opioid receptor and mGluR5, specifically forming a μ‐opioid receptor–mGluR5 heteromer. This heteromer is co‐localized in the spinal dorsal horn and glial cells [125]. The presence of this heteromer, along with findings that MPEP can enhance the analgesic effect of morphine and inhibit morphine‐induced tolerance and dependence, suggests potential design strategies for developing novel analgesics that target μ‐opioid receptors and mGluRs [126, 127, 128].

## 5 Groups II and III mGluRs

Multiple group II and III mGluRs are expressed on pre‐ and postsynaptic elements of the dorsal root ganglion somata and peripheral nerve terminals in the dorsal horn [129]. Despite their widespread expression in central and peripheral pain pathways, studies of the roles of group II and group III mGluRs in morphine tolerance are limited.

LY354740, the first systemically active group II mGluR agonist, attenuated the development of tolerance to the analgesic effects of morphine in the tail‐flick test in adult mice [130]. Targeting presynaptic mGluR activation at nerve terminals has been demonstrated to reduce glutamate release both in vitro [131] and in vivo [132]. The mechanism by which group II mGluR agonists inhibit morphine tolerance may also involve decreasing the function of the NMDA receptor through inhibition of glutamate release [133]. Another probable mechanism underlying the reversal of morphine tolerance development by LY354740 may involve inhibition of the cyclic AMP (cAMP) upregulation system. These effects may be the basis of opiate dependence and tolerance [134].

Administration of AMN082, a selective mGluR7 allosteric agonist, inhibited the development of morphine tolerance. In addition, a bolus injection of AMN082 into tolerant mice successfully reversed the analgesic tolerance of morphine [135]. MMPIP, a metabotropic glutamate receptor 7 antagonist, reversed the effects of AMN082 on morphine tolerance. Because of its presynaptic location, activation of group III metabotropic glutamate receptors can inhibit NMDA receptor activity [136] and thereby reduce NMDA‐mediated morphine tolerance.

## 6 Crosstalk Between mGluR5 and NMDAR in Morphine Tolerance

**FIGURE 4:**
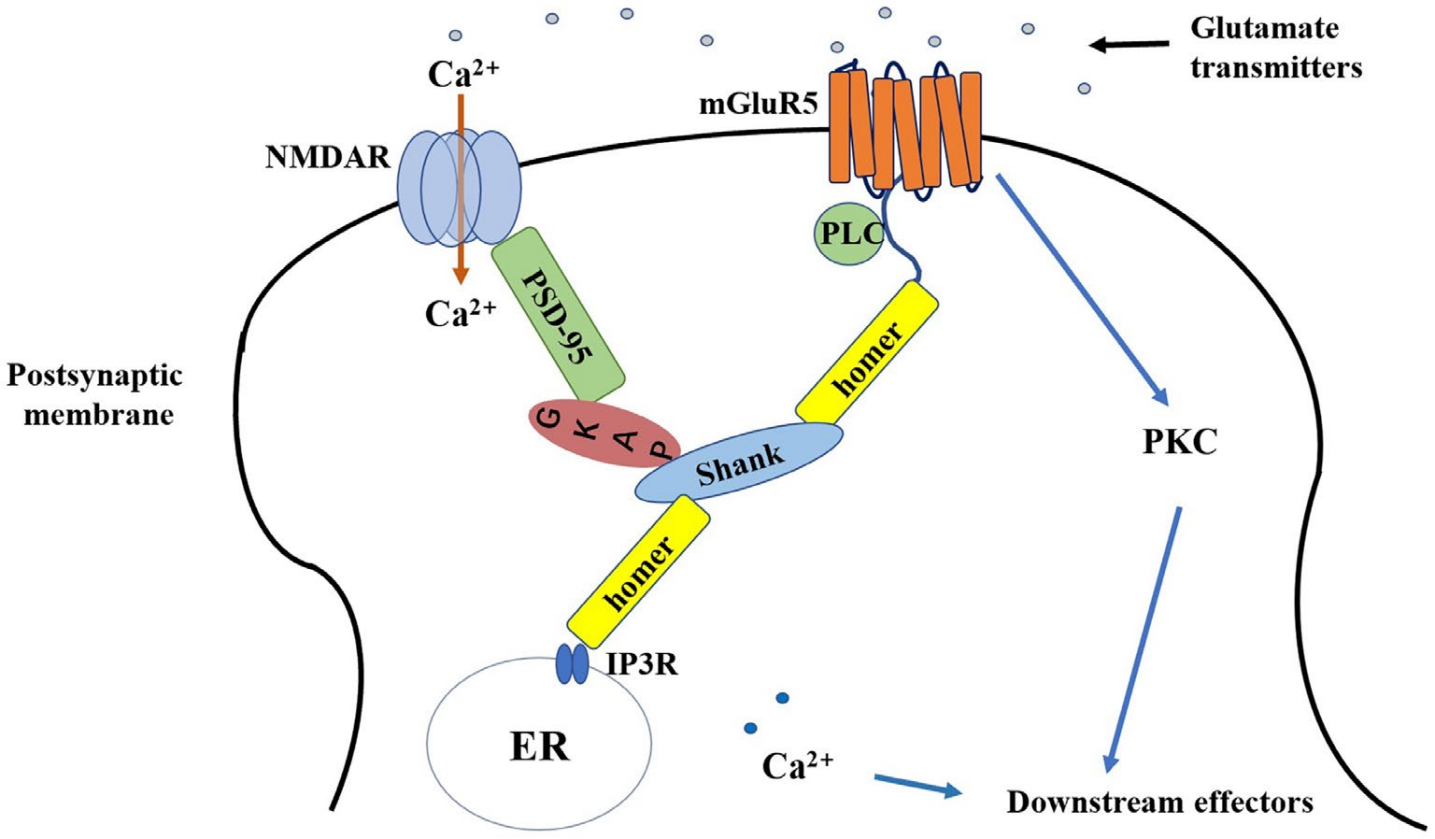
Schematic diagram of NMDA receptor and metabotropic glutamine receptor 5 connections, including some related PSD proteins.

Several known structural proteins link and regulate the activation, localization, and signaling of mGluR5 and NMDA receptors (Figure 4) [137]. One of the most pronounced effects of mGluR activation in numerous regions of the CNS is the enhancement of agonist‐evoked currents through NMDA receptor cation channels [138, 139, 140].

**FIGURE 5:**
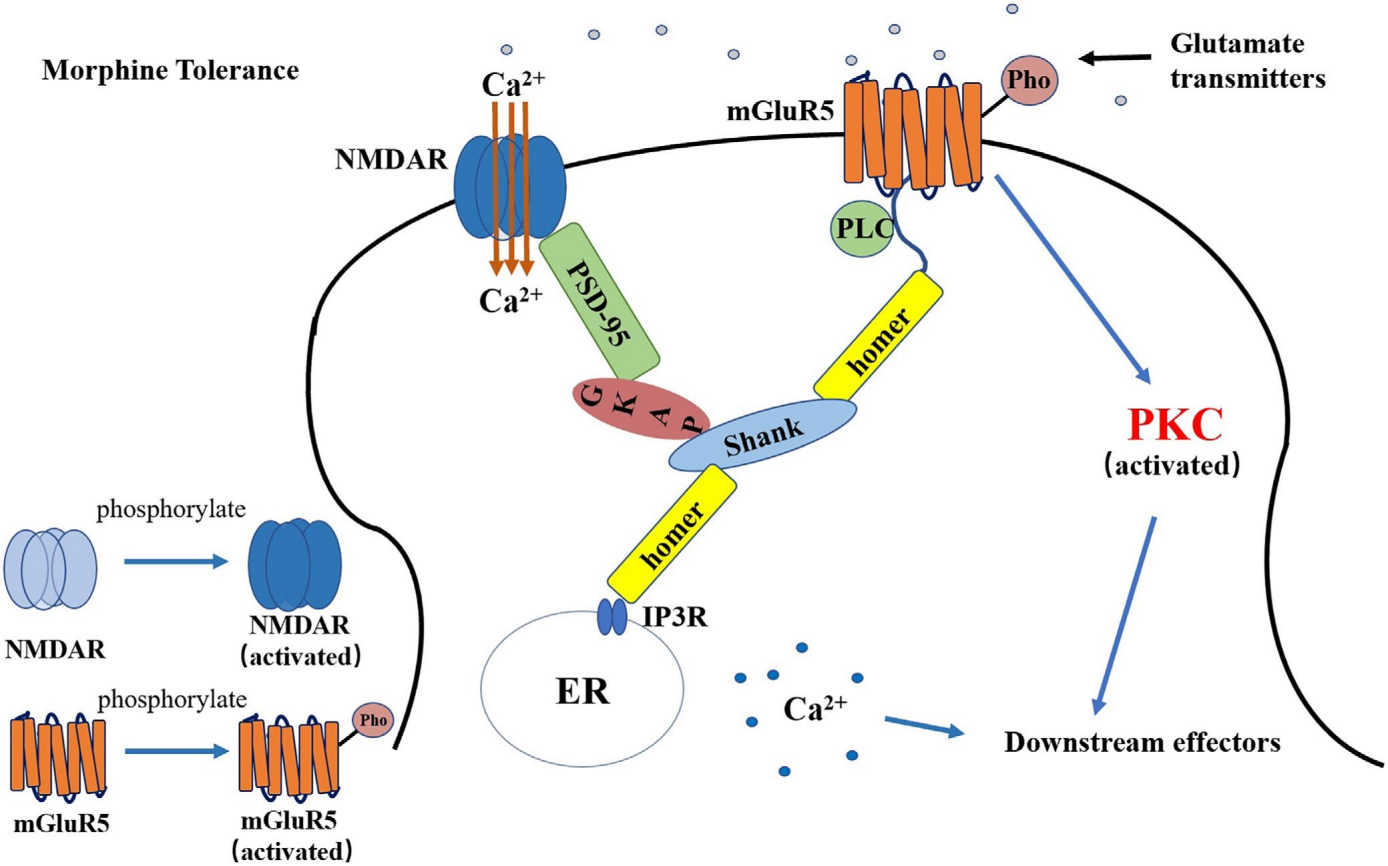
A schematic diagram of PSD protein connections after morphine tolerance.

The interaction between mGluR5 and NMDA receptors during the development of morphine analgesic tolerance has not been extensively explored. Our previous research demonstrated that the physiological connection between mGluR5 and NMDA receptors through PSD proteins may not play a significant role in NMDA receptor‐mediated regulation of morphine tolerance [121, 124, 141]. In contrast, mGluR5‐mediated PKC signaling may play a more crucial role in the mechanisms underlying morphine tolerance [121, 124, 141] (Figure 5).

mGluR5 increases the phosphorylation of the NMDA NR1 receptor [67]. In addition, the increased NR1 level caused by chronic morphine treatment was inhibited by antagonizing the activation of mGluR5 [68]. In a recent study, MMG22, a bivalent ligand‐containing MOR agonist and mGluR5 antagonist, was used to inhibit NMDA receptor‐mediated hyperalgesia by antagonizing mGluR5 while activating mu‐opioid receptors to enhance the antinociceptive effect [128].

Recently, another study showed that repeated treatment with opiates such as morphine and fentanyl reduced monomeric mGluR5 protein levels in the DRG but increased levels of mGluR5 monomers and homodimers in the spinal cord in mice and rats of both sexes. In the spinal cord, mGluR5 proteins dimerize and physically interact with NMDARs to augment their synaptic expression and activity. Through dynamic interactions, the two distinct glutamate receptors mutually amplify and sustain nociceptive input from peripheral sensory neurons to the spinal cord. Thus, inhibiting mGluR5 activity or disrupting mGluR5‐NMDAR interactions could reduce opioid‐induced hyperalgesia and tolerance and potentiate opioid analgesic efficacy (doi: 10.1523/JNEUROSCI.0601‐23.2023).

## 7 Therapeutic Potential

Scientists have long been interested in regulating excitatory glutamate receptors to interfere with the progression of morphine tolerance. Identifying additional links between morphine tolerance and the modulation of excitatory glutamate receptors is necessary to improve our understanding of drugs that specifically modulate excitatory glutamate receptors to restore the analgesic effects of morphine. Here, we summarize the information on several drugs that have been shown to delay the onset of morphine tolerance by regulating excitatory glutamate receptors and have been approved for clinical use.

The following are well‐studied drugs that regulate excitatory glutamate receptors, including several approved for clinical use, with mechanisms that may be useful, and elucidating their possible mechanisms in the process of influencing morphine tolerance would be expedient.

### 7.1 Ketamine

Ketamine is a commonly used NMDA receptor antagonist in clinical practice. In addition to its anesthetic effects, it has analgesic, anti‐inflammatory, and antidepressant effects, despite side effects such as dissociative symptoms and psychological symptoms [86]. Ketamine can be used for analgesia in patients with opioid tolerance [142, 143, 144]. It is reported that β‐arrestin activity was increased by the combination of ketamine with fentanyl, which is associated with improved desensitization of MOR, and ERK might also be activated through this process [145]. However, whether this modulation occurs directly at the receptors or through downstream signals, and its effect on receptor desensitization, requires further research for clarification.

### 7.2 Donepezil

Donepezil is a reversible, noncompetitive acetylcholinesterase inhibitor with some efficacy in patients with mild to moderate Alzheimer's disease [6]. Donepezil prevents neuronal apoptosis and attenuates morphine tolerance [146]. Moreover, donepezil reportedly inhibited chronic morphine‐induced overexpression of NR1 in cancer pain in rats, which was associated with the reduction of glutamine toxicity by downregulation of NMDA receptors, especially NR1 levels [147]. However, chronic morphine treatment upregulated NR2, but not NR1 [124]. We speculate that the conflicting results may be due to varying expression levels of NMDA receptors in different regions in different animal models.

### 7.3 Melatonin

Melatonin is a pineal neurohormone that plays important roles in the biological regulation of various physiological and pathological activities, including circadian rhythms, sleep, mood, reproduction, tumor growth, and neuroprotection [148, 149]. It is also one of the most widely used sleep‐regulating medicines. Melatonin can inhibit the upregulation of PKCγ and NR1, thereby alleviating the decline in the percentage of maximum possible antinociceptive effects of morphine (MPAE%) and preventing the development of tolerance in rats treated with long‐term morphine [23]. Systemic and intrathecal administration of melatonin produce dose‐dependent analgesic effects and also prevent chronic morphine‐induced hyperalgesia [150, 151]. Recently, studies from Gobbi lab showed that an interneuronal circuit between melatonin and the opioid system, that is, melatonin MT_2_ receptor agonism requires MORs to exert its antiallodynic effects [152]. However, further studies are required to elucidate the specific cellular mechanisms involved.

### 7.4 Allosteric Modulators or Bivalent Ligands Targeting Opioid and Glutamate Receptors

Allosteric modulators are thought to play key roles in influencing receptor functions, such as tolerance to ligands, and affecting downstream pathways. Many efforts have been made to explore the role of allosteric modulators or bivalent ligands targeting opioid and glutamate receptors in the development of morphine tolerance. Here, we will give a brief summary.

The MOR/DOR heterodimer is present in regions of the CNS that are thought to be important in the processing of addiction and tolerance induced by long‐term morphine administration [153]. It has also been shown that long‐term administration of morphine and various protein chaperones upregulates MOR/DOR, leading to changes in the binding characteristics and functional signaling of the heteromer [154, 155]. Therefore, we can design novel drugs targeting PAM dedicated to DOR to enhance MOR agonist signaling, thereby providing better analgesia and fewer serious adverse effects. This has the advantage that such ligands do not interact with MOR or MOR homodimers, thus increasing selectivity in the presence of MOR/DOR heterodimers alone [156].

A novel GluN2B‐selective negative allosteric modulator, EU93‐108, showed high potency and brain penetrance. It has an acute and significant anodyne effect and can enhance the analgesic effect of morphine. The experimental data also suggested that engagement of GluN2B as a target has utility in the treatment of pain, and EU93‐108 could serve as a suitable tool compound to interrogate this hypothesis. Future structure–activity relationship work around this scaffold could give rise to compounds that can be co‐administered with opioids to diminish the onset of tolerance due to chronic opioid use, thereby modifying their utility [157].

## 8 Conclusion

Morphine remains a staple for managing moderate to severe pain and is extensively used clinically. The mechanisms underlying the development of tolerance to morphine continue to garner considerable global attention. In this review, we specifically describe the important research results and progress of ionic excitatory glutamate receptors represented by NMDA receptors and metabotropic excitatory glutamate receptors represented by I mGluRs in the development of morphine tolerance. Since excitatory glutamate receptors play an important role in the development and progression of morphine tolerance, drugs targeting these receptors have received extensive attention from scientists. In this review, we also collate some marketed drugs that target the action of excitatory glutamate receptors, as well as drugs in development that target allosteric modulators or bivalent ligands of opioid and glutamate receptors. While the development of drugs targeting these receptors is ongoing, their clinical application is currently limited, primarily due to their extensive effects on the CNS. Nonetheless, the potential future role of such agents in clinical practice appears promising.

## Author Contributions

X.M. and T.X.: conceptualization, writing – reviewing, and editing. M.H., L.L., W.W., and H.X.: writing – original draft preparation. M.C.: supervision. All authors have approved the final version of the manuscript to be submitted.

## Conflicts of Interest

The authors declare no conflicts of interest.

## Data Availability

Data sharing not applicable to this article as no datasets were generated or analysed during the current study.
